# Endothelial Dysfunction in Pregnancy Complications

**DOI:** 10.3390/biomedicines9121756

**Published:** 2021-11-24

**Authors:** Jakub Kornacki, Paweł Gutaj, Anastasia Kalantarova, Rafał Sibiak, Maurycy Jankowski, Ewa Wender-Ozegowska

**Affiliations:** 1 Department of Reproduction, Poznan University of Medical Sciences, 33 Polna Street, 60-535 Poznan, Poland; kuba.kornacki@wp.pl (J.K.); ewaoz@post.pl (E.W.-O.); 2 Medicine Program, Poznan University of Medical Sciences, 41 Jackowskiego Street, 60-512 Poznan, Poland; 80623@student.ump.edu.pl; 3 Department of Histology and Embryology, Poznan University of Medical Sciences, 6 Swiecickiego Street, 60-781 Poznan, Poland; rafal.sibiak@student.ump.edu.pl; 4 Department of Anatomy, Poznan University of Medical Sciences, 6 Swiecickiego Street, 60-781 Poznan, Poland; mjankowski@ump.edu.pl

**Keywords:** endothelial dysfunction, endothelium, preeclampsia, FGR, diabetes

## Abstract

The endothelium, which constitutes the inner layer of blood vessels and lymphatic structures, plays an important role in various physiological functions. Alterations in structure, integrity and function of the endothelial layer during pregnancy have been associated with numerous gestational complications, including clinically significant disorders, such as preeclampsia, fetal growth restriction, and diabetes. While numerous experimental studies have focused on establishing the role of endothelial dysfunction in pathophysiology of these gestational complications, their mechanisms remain unknown. Numerous biomarkers of endothelial dysfunction have been proposed, together with the mechanisms by which they relate to individual gestational complications. However, more studies are required to determine clinically relevant markers specific to a gestational complication of interest, as currently most of them present a significant overlap. Although the independent diagnostic value of such markers remains to be insufficient for implementation in standard clinical practice at the moment, inclusion of certain markers in predictive multifactorial models can improve their prognostic value. The future of the research in this field lies in the fine tuning of the clinical markers to be used, as well as identifying possible therapeutic techniques to prevent or reverse endothelial damage.

## 1. Introduction

The endothelium is a unique monolayer of cells lining the blood vessels. It was once believed to be a rigid structure with a protective purpose only. In turn, it is now considered to play a role in many biological functions including vasomotor tone, hemostatic balance, cell trafficking, permeability, proliferation, survival and immunity. In addition to the multifunctional nature of the endothelium, it is also characterized by phenotypic heterogeneity. For example, postcapillary venule endothelial cells are mainly responsible for leukocyte trafficking, while the arteriolar wall endothelium primarily regulates motor tone [1].

Endothelial cells of the vascular system are covered by a carbohydrate polymer known as the endothelial glycocalyx (EG), which maintains tissue integrity, prevents leukocyte and platelet adhesion, and presents antithrombotic activity [2]. Located on the luminal surface of endothelial cells, EG is a polyanionic structure consisting of membrane anchored glycosphingolipids, glycoproteins, proteoglycans and glycosaminoglycans (GAGs) [3]. Proteoglycans, such as syndecans, glypicans and endocans, are the core transmembrane proteins that exhibit carbohydrate attachments in the form of GAG chains (heparan sulphates, chondroitin sulphates, dermatan sulphates). Heparan sulphates are the most abundant type of GAG within the EG, accounting for 50–90% of all proteoglycans. In contrast with other glycocalyces, EG is denser, ranging between 0.2 and 0.5 μm in capillaries and 2–3 μm in small arteries. EG is believed to play a role in multiple functions associated with vascular processes, including vascular permeability, inflammation, thrombosis, mechanotransduction, and cytokine signalling [4]. Moreover, EG damage was reported to result in edema and albuminuria [5].

Recently, a glycocalyx-like structure has been found on the surface lining of placental blood vessels [6]. This structure, referred to as the placental glycocalyx, is produced by syncytiothrophoblasts as a part of the extracellular matrix of maternal blood vessels. Syncytiotrophoblasts are believed to fuse together into a continuous layer of cells forming the exterior surface of chorionic villi. Due to the epithelial origin and unusual location of syncytiotrophoblasts, it is possible that the nature of glycocalyx would be different from that of endothelial cells [4]. With that said, there are only a few studies that analyzed the composition of the placental glycocalyx in detail, and those that did found some similarities to EG [7]. Both placental and vasculature endothelia are abundantly covered by syndecans. Although the role of the placental glycocalyx is still a point of investigations, alterations in its structure, along with endothelial dysfunction, have been implemented in preeclampsia (PE) [8].

Overall, injury to the endothelium and associated structures has been implicated in many gestational complications, including PE, fetal growth restriction (FGR), and diabetes. The aim of this article is to review and discuss the proposed mechanisms by which endothelial dysfunction is implicated in pregnancy complications, as well as to identify common mechanisms and responsible biomolecules. We believe that this knowledge may in the future assist in differentiating some of these pathologies and provide important clinical information about the severity of the disease.

## 2. Methods

**Figure 1:**
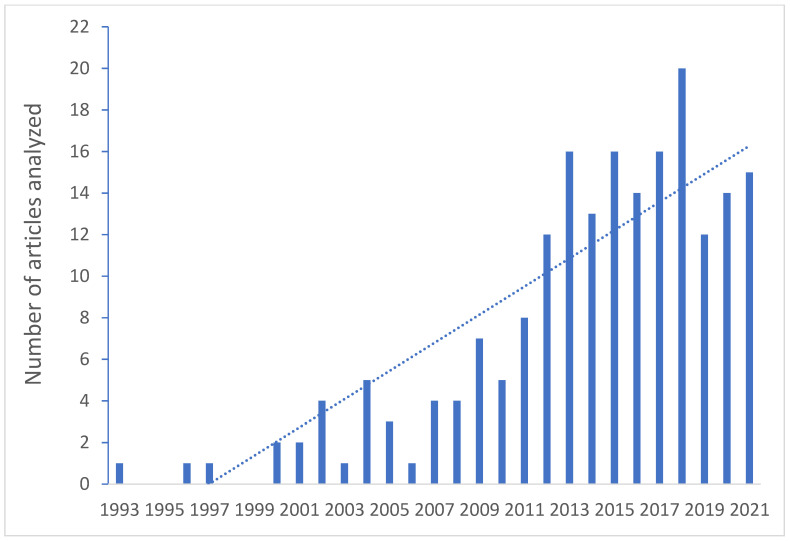
Bar chart showing the number of publications published in certain years that were analyzed and included as part of this review article.

To compile this narrative review, we searched the PubMed database for relevant references up to November 2021 using the following terms: “endothelial dysfunction preeclampsia”, “endothelial dysfunction gestational hypertension”, “endothelial dysfunction fetal growth restriction”, “endothelial dysfunction intrauterine growth restriction”, “endothelial dysfunction diabetes pregnancy”, and “endothelial dysfunction gestational diabetes”. We also searched the database with all the phrases mentioned above using the terms “endothelial damage” and “endothelial injury” instead of “endothelial dysfunction”. Bibliographies from included articles were reviewed for the purpose of identifying any additional relevant articles that aligned with the objective of the paper. We initially excluded articles not related to the aims of the review, analyzing the titles and abstracts. Then, we analyzed the full texts of each paper included in this review. We selected only the articles written in English during the manuscript preparation, which could be a possible study limitation. Distribution of articles that were analyzed as part of the review is depicted in Figure 1.

## 3. Discussion

### 3.1. Preeclampsia (PE)

PE is probably the most characteristic pregnancy complication associated with endothelial injury and dysfunction. This association includes the two most important issues: (1) a predisposition to PE in pregnant women with a primary endothelial dysfunction and (2) a secondary endothelial injury as a consequence of a primary impaired placental perfusion, the two main phenomena in the pathophysiology of PE [9,10,11].

**Figure 2:**
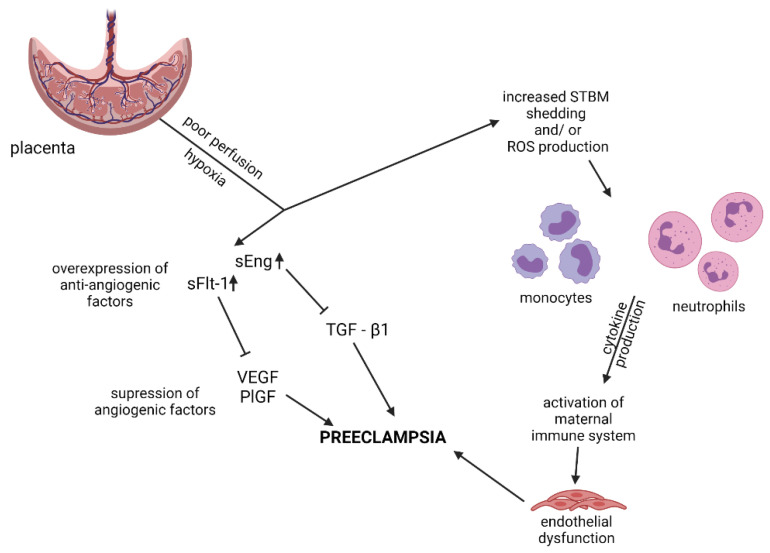
The possible mechanisms of PE pathogenesis. sEng-soluble endoglin; sFlt-1-soluble fms-like tyrosine kinase 1; VEGF-vascular endothelial growth factor; PIGF-placental growth factor; TGF-β1-transcription growth factor β1; STBM-syncytiotrophoblast microparticles; ROS-reactive oxygen species. Created with Biorender.com (accessed on 19 October 2021).

**Figure 3:**
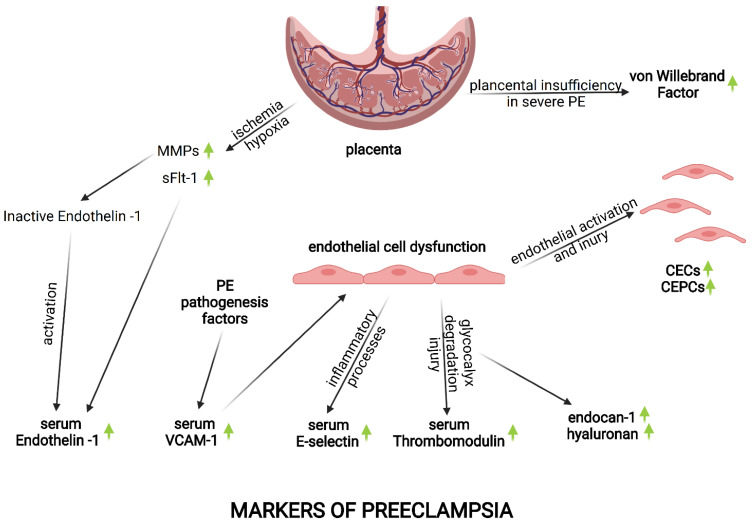
Markers of preeclampsia and their implication in its pathogenesis and progression. MMPs-matrix metalloproteinases; sFlt-1-soluble fms-like tyrosine kinase 1; VCAM-1-vascular cell adhesion molecule 1; PE-preeclampsia; CECs-circulating endothelial cells; CEPCs-circulating endothelial progenitor cells. Created with Biorender.com (accessed on 19 October 2021).

The most important factor contributing to endothelial injury in PE is an increased production of antiangiogenic factors, including soluble fms-like tyrosine kinase 1 (sFlt-1) and soluble endoglin (sEng), by a poorly perfused, hypoxic placenta (Figure 2) [9,12]. SFlt-1, in case of overproduction, neutralizes vascular endothelial growth factor (VEGF) and placental growth factor (PlGF), which are well known protectors of vascular and endothelial structures (Figure 2 and Figure 3) [10,12,13]. Similarly, by binding and neutralizing soluble transforming growth factor-β1 (TGF-β1) in the blood, sEng contributes to increased vascular permeability (Figure 2) [13].

Another possible mechanism of endothelial dysfunction in PE involves activation of the maternal immune system caused by an increased production of cytokines by different types of leukocytes, including monocytes and neutrophils [14,15,16]. Both cytokines and neutrophils (directly) may contribute to endothelial injury [14]. Among possible mechanisms which possibly result in activation of immune systems in patients with PE are: (1) the increased shedding of syncytiotrophoblast microparticles (STBM) by poorly perfused placenta, and (2) the increased production of reactive oxygen species (ROS) in hypoxic placenta (Figure 2) [14,16].

The significance of endothelial dysfunction in PE is especially important from a clinical point of view, because most of the clinical symptoms of PE, especially the maternal ones, may be explained by this phenomenon [11,17,18]. The most characteristic clinical consequences of endothelial damage are: (1) proteinuria, (2) oedema, and (3) hypertension. Another interesting clinical aspect is the presence of a possible difference in the degree of endothelial damage in patients with early- (EOP) and late-onset PE (LOP).

In PE, similarly to other clinical pathologies, the presence and the degree of endothelial dysfunction may be assessed by different methods, which include non-invasive in vivo and in vitro methods.

#### 3.1.1. Methods of Endothelial Dysfunction Assessment in PE

The gold standard of non-invasive in vivo methods is flow-mediated dilation (FMD), and its modified version, known as reactive hyperemia peripheral arterial tonometry (RH-PAT) [19,20,21].

In comparison to healthy pregnant women, a significantly impaired endothelial function, manifesting in a reduction of FMD, was found in patients with PE [19,22]. These differences were most significant in the third trimester of pregnancy, especially after around 30 weeks of gestation, when using both the FMD [19] and the RH-PAT methods [21]. However, impaired endothelial function in women with PE compared to the healthy pregnant ones could already be observed in the second trimester of pregnancy, several weeks before the development of clinical symptoms of PE [22].

Some controversies concern the results of endothelial function after delivery in women who developed PE. Whereas an earlier meta-analysis [22] showed a lower value of FMD three years after delivery in women with previous PE compared to the control group, another study [23] indicated a quick normalization of these results, even after one month post-partum.

Interestingly, so far, there are no comparative studies on the degree of endothelial dysfunction assessed by these non-invasive in vivo methods in early- (EOP) and late-onset PE (LOP). There is also no data on the association of such studies with clinical results and characteristics of patients with PE.

The in vitro assessment of endothelial function/dysfunction consists of the measurement of the serum concentration of different markers of endothelial dysfunction. The most common include: (1) endothelin-1 (ET-1), (2) vascular adhesive molecule-1 (VCAM-1), (3) selectins (4) thrombomodulin, (5) markers of endothelial glycocalyx (EG) degradation, (6) the von Willebrand factor, (7) circulating endothelial cells (CECs), and circulating endothelial progenitor cells (CEPCs).

#### 3.1.2. Endothelin-1

Endothelin-1 is known as a strong vasoconstrictor produced mainly by endothelial cells [24]. In numerous previous PE studies, ET-1 serum levels were found to be significantly elevated compared to healthy pregnant women [25,26,27,28]. Interestingly, serum ET-1 was also found to be significantly higher, even in the first trimester of pregnancy, in women who later developed PE compared to those who remained normotensive [29]. Serum levels of ET-1 were especially increased in women who developed the HELLP syndrome, being even higher than in preeclamptic patients without HELLP [30]. The specific genetic predisposition of the Lys198Asn polymorphism affected patients for increased ET-1 serum concentration was also found [31]. Moreover, ET-1 levels were also found to be significantly higher in placental tissues from patients with PE compared to those of healthy pregnant women [32].

**Figure 4:**
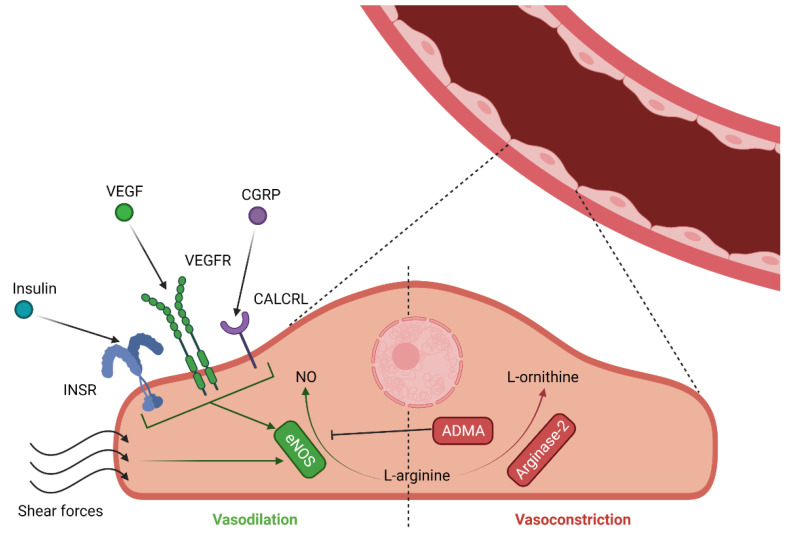
Selected vasculature related molecular mechanisms implicated in FGR and PE. INSR-insulin receptor; VEGF-vascular endothelial growth factor; VEGFR-VEGF receptor; CGRP-calcitonin gene-related peptide; CALCRL-calcitonin receptor-like receptor; NO-nitric oxide; eNOS-endothelial nitric oxide synthase; ADMA-asymmetric dimethylarginine. Created with Biorender.com (accessed on 19 October 2021).

While the exact mechanism of increased ET-1 production in women with PE is unknown, it could be a consequence of increased production of sFlt-1 and matrix metalloproteinases (MMPs) in ischemic, hypoxic placentas (Figure 4) [33]. Animal studies revealed that MMPs may be responsible for the cleavage of big-ET-1 to an active ET-1 form [33]. A significant correlation between serum concentrations of ET-1 and sFlt-1, as well as ET-1 and the sFlt-/PlGF ratio, was described by Aggarwal et al. in women with PE [34]. Interestingly, one of the statins (pravastatin) significantly reduced secretion of both ET-1 and sFlt-1 in the in vitro model [35].

#### 3.1.3. VCAM-1

The key pathophysiological element of endothelial dysfunction is its activation, which may be triggered by different factors, including those involved in the pathogenesis of PE. Serum levels of the soluble form of VCAM-1 (sVCAM-1) are a known marker of endothelial activation (Figure 3). PE is characterized by an increased serum concentration of s-VCAM-1, significantly higher than in normal pregnancy [11,36,37,38]. However, some controversies concern the difference in the levels of sVCAM-1 in patients with EOP and LOP. In the earlier study, sVCAM-1 was found to be lower in LOP compared to EOP [39], whereas more recent studies did not show such a difference [11,40]. Moreover, the rs3181092 polymorphism of VCAM-1 was found to be associated with an increased risk of PE [41].

Interestingly, in one of above-mentioned studies [36], a significant negative correlation was observed between sVCAM-1 level and birth weight and gestational age at delivery, which emphasizes the utility of such measurement. Another interesting finding was a decreased expression of VCAM-1 observed in the placentas of women with PE in comparison to healthy ones, which may indicate another role of VCAM in the placenta, for example, in the process of placentation [42].

#### 3.1.4. E-Selectin, P-Selectin, L-Selectin

Selectins are transmembrane proteins produced by the endothelium (E-selectin), platelets (P-selectin), and leukocytes (L-selectin), which are activated and translocated to the cell surface upon activation of the inflammatory process associated, among others, with increased migration and adhesion of different immune cells [43,44]. Interestingly, selectins also play a role in the process of implantation [45].

The most indicative marker of endothelium damage and dysfunction seems to be the soluble E-selectin (sE-selectin) (Figure 3) [43]. Increased serum concentration of sE-selectin in patients with PE in comparison to normotensive pregnant women was identified in multiple studies [37,43,46,47,48]. Interestingly, in the study by Papakonstantinou et al. [46] these differences were observed only in patients with PE, and not in patients with gestational hypertension. On the other hand, Mistry et al. [43] observed a significantly higher level of sE-selectin in patients with EOP compared to patients with LOP, which indicates a correlation between levels of this marker and severity of the disease. Additionally, Mehrabian et al. [48] and Carty et al. [49] found an increased level of sE-selectin many weeks before the development of PE, including in the first trimester of pregnancy. Unlike sE-selectin, the serum concentrations of soluble P-selectin (sP-selectin) and soluble L-selectin (sL-selectin) do not seem to be as reliable markers of endothelial dysfunction and damage in PE as sE-selectin. In turn, while sP-selectin serum levels were found to be increased in patients with PE in some previous studies [37,50], it was not confirmed in the latest one by Mistry et al. [43]. Moreover, conflicting results have been reported regarding the concentration of sL-selectin in patients with PE [37,43].

#### 3.1.5. Thrombomodulin (TM)

Thrombomodulin (TM) is a transmembrane glycoprotein expressed mainly on the glycocalyx surface of endothelial cells [51]. It has an important role in the process of hemostasis, inflammation and apoptosis (Figure 3) [51]. Its anticoagulation activity is a consequence of protein C activation and thrombin deactivation [52,53]. Therefore, an endothelial injury with a degradation of its glycocalyx structure may contribute to intravascular hypercoagulability, which is a PE characteristic.

Another interesting role of TM may concern the maintenance of the glomerular filtration barrier, which has been proved in diabetic mice [51].

Increased serum concentrations of TM in patients with PE in comparison to normotensive pregnant women is probably the consequence of its cleavage associated with endothelial glycocalyx degradation, as was reported by Minakami et al. [54] in 1993. Lately, these findings have been confirmed by Zhu et al. [55] and Alpoim et al. [56]. Zhu et al. [55] recorded increased levels of TM in both EOP and LOP patients, whereas the second study [56] only observed an increase of TM in patients with severe EOP.

There are more controversies concerning the predictive value of TM level assessment in pregnant women. Conflicting results in this field were obtained by Prochazka et al. [57] and Wang et al. [58], both of which do not consider measurement of TM clinically relevant in the prediction of PE.

So far, only two studies have measured mRNA expression of TM in placental tissues [55,59]. While both studies have identified decreased mRNA expression of TM in placentas of PE patients, in a study by Zhu et al. [55] this finding concerned mainly EOP.

The only study on expression of TM in kidneys of patients with PE was done by van Aanhold et al. [51]. The authors found increased glomerular TM mRNA expression in patients with PE compared to the control group. This observed upregulation, according to authors of the study, may have a protective role.

#### 3.1.6. Markers of Endothelial Glycocalyx (EG) Degradation

The endothelial glycocalyx (EG) is the most important protective, external structure of endothelium. It is an external layer of endothelial cells composed of different proteoglycans (PGs), glycoproteins, glycolipids and GAGs [2,60]. The protective role of EG for endothelium includes, among others, maintenance of tissue integrity, prevention of leukocytes and platelets adhesion, and antithrombotic activity [2,60].

Moreover, serum concentrations of soluble components of EG may reflect the degree of endothelial damage as a consequence of the shedding of EG components.

The most reliable markers of EG degradation seem to include endocan-1 (ESM-1), hyaluronan (HA) and syndecan-1 (SDC-1) (Figure 3).

Endocan-1 is one of the important PGs of EG. In most of the studies, the serum level of ESM-1 was found to be significantly increased in patients with PE, compared to the normotensive pregnant women [61,62,63]. However, in two other studies [64,65], including the most recent one [65], the serum concentration of ESM-1 did not differ between patients with PE and the control group. Important findings were described by Adekola et al. [54], who found a significant correlation between the concentration of ESM-1 and the levels of antiangiogenic factors, including sFlt-1 and sEng. Additionally, no difference concerning the serum concentration of ESM-1 was found between patients with EOP and LOP. On the other hand, Cakmak et al. [63] found some positive correlations between the serum level of ESM-1 and some clinical characteristics, including the value of systolic and diastolic blood pressure and the degree of proteinuria.

Hyaluronan is an important GAG component of EG. Interestingly, so far, it seems to be the most reliable marker of EG degradation, indicating both endothelial dysfunction and activation (Figure 3). So far, in few, but all available studies, the significantly higher serum level of HA was found in patients with PE than in healthy pregnant women [11,22,66,67,68,69]. Similar to the study by Adekola et al. [61], which discussed ESM-1, Kornacki et al. [3,59] did not report a significant difference in the level of HA in patients with EOP and LOP. Both of these studies indicate a common pathophysiological mechanism of EOP and LOP, including the comparable degree of endothelial damage in both types of PE. In the another study by Kornacki et al. [67], while no significant correlation was generally found between the level of HA and the concentration of sFlt-1, such a trend was observed only for patients with EOP.

Another practical use of serum HA level assessment was proposed by Wiles et al. [70], who found that an increased level of HA was a good marker differentiating between PE and exacerbation of the primary condition in patients with diabetic kidney disease. In those who developed PE, the concentration of HA, as well as sVCAM-1, was significantly higher [70].

Syndecan-1, like ESM-1, is an important PG of EG. Interestingly, most of the available data indicate the presence of lower serum levels of SDC-1 in patients with PE than in normotensive pregnant women [66,71,72]. Only Lahsinoui et al. [67] and Weissgerber et al. [22] did not find significant differences between such levels in patients with PE and those from the control groups. These findings of rather low serum levels of SDC-1 in patients with PE are in contrast with the opposite data on the concentrations of ESM- 1 and HA. This may indicate another significant source of SDC-1 during pregnancy, which could be the placenta [22,73]. Also, SDC-1 is universally increased in all pregnant women, and it has a positive correlation with gestational age, which decreases its utility in indicating endothelial damage [74].

#### 3.1.7. Von Willebrand Factor (vWf)

The Von Willebrand Factor (vWf) is a glycoprotein produced by endothelial cells and megakaryocytes. It plays an important role in vascular hemostasis, including promoting the adhesion of platelets to the endothelium and stabilizing of Factor VIII [75]. Additionally, it is involved in the process of inflammation, being released into circulation by activated endothelial cells (Figure 3) [44,75]. All of the above factors make the serum concentration of vWf one of the endothelial activation and injury markers [44].

In recent years, there is limited data on the assessment of the serum concentration of vWf in patients with PE. In two of the studies [76,77], a significantly increased serum level of vWf was found in patients with PE compared to the control group, but only in the case of a presence of placental insufficiency and in severe PE. In the two other studies, the concentration of vWf did not differ statistically between the patients with PE and normotensive pregnant women [57,78].

#### 3.1.8. Circulating Endothelial Cells (CECs) and Circulating Endothelial Progenitor Cells (CEPCs)

Both CECs and CEPCs may be used as another blood marker of endothelial injury and dysfunction [44]. In the process of endothelial activation and injury, these cells may be detached and released in a higher amount into the circulation [44,78]. The mechanisms that may cause increased shedding of CECs include apoptosis, mechanical injury, an imbalance between pro- and antiangiogenic factors, weakening of the intracellular connections, endothelial structure injury by cytokines and proteases, as well as the activity of different drugs (Figure 3) [78].

Both types of cells may be identified in the blood using multicolour flow cytometry or immunomagnetic techniques. CECs, as opposed to CEPCs, are CD-133 negative, since they lose this antigen during maturation [79]. Hence, the CEPC/CEC ratio is another potential marker of endothelial activation and dysfunction [80].

In a few recently published studies, women with PE were found to have an increased number of CECs in serum compared to healthy controls [48,78,81]. At the same time, Lagana et al. [82] found a decreased number of CEPCs in patients with PE compared to normotensive pregnant women. In turn, Szpera et al. [78] and Heimrath et al. [83] observed decreased CEPCs only in patients with chronic hypertension and gestational hypertension.

Additionally, Mehrabian et al. [48] observed an increased number of CECs in patients with PE even a few weeks prior to the onset of disease, which emphasizes the role of these cells in predicting PE.

Finally, both patients with PE and those with chronic hypertension in pregnancy had a significantly decreased CEPC/CECs ratio compared to healthy pregnant women [78].

### 3.2. Fetal Growth Restriction

FGR is a condition in which the fetus does not achieve full growth potential in utero due to genetic or environmental factors. Clinically, FGR is diagnosed when estimated fetal weight is below the 10th percentile for the gestational age. There can be multiple factors contributing to FGR, including maternal, fetal and placental. These causes can also overlap with each other; therefore, a clear differentiation between them is often complex. FGR is associated with increased fetal and neonatal morbidity and mortality. Moreover, it is reported that neonates with FGR face an increased risk of atherosclerosis, hypertension, coronary artery disease and chronic kidney disease in the future [84]. While the endothelium plays a crucial role in maintaining a proper vascular function and homeostasis both prenatally and postnatally, it is not surprising that its dysfunction can lead to long-term cardiovascular-related disorders.

In this section, we will focus mainly on the placental origin of FGR, as it is believed to involve endothelial dysfunction in its pathomechanism [85]. Impaired vascular remodeling and decreased vascular volume affect placental vascularity and the capability to respond to vasodilatory signals. The combination of these changes leads to decreased oxygen supply to the fetus, which results in chronic hypoxia, contributes to reduced growth of the fetus, and causes the development of an oxidative stress environment [86,87].

#### 3.2.1. Balance between Vasodilation and Vasoconstriction Signals in of FGR Placental Vessels

It is believed that morphology and function of the vasculature of various levels (i.e., arteries and veins) can be modified by environmental factors, such as blood flow, levels of oxygen, epigenetic factors and the level of oxidative stress [88]. Nitric oxide (NO), produced by endothelial nitric oxide synthase (eNOS), is believed to be functionally related to these environmental factors (Figure 4) [89]. The release of NO is triggered by either shear stress or, to a lower extent, through binding of insulin, vascular endothelial growth factor (VEGF) and calcitonin gene-related peptide (CGRP) (Figure 4) [90,91,92]. Increased levels of NO-related metabolites were described in FGR and PE placentas [93]. Similarly, eNOS, the activity of which results in NO generation, exhibited significantly increased expression in chorionic and umbilical arteries, and decreased levels in the umbilical vein in FGR [94,95,96]. Umbilical vein findings were also confirmed during in vitro experiments on FGR-derived human umbilical vein endothelial cell (HUVEC) cultures [97]. The vasodilatory activity of eNOS is believed to be counteracted by arginase-2 (ARG2), since both of them compete for the same substrate, L-arginine (Figure 4). When it comes to FGR, ARG2 expression and activity were increased in cultured endothelial cells from FGR umbilical arteries (HUAEC), but not veins (HUVEC) [93]. At the same time, decreased in vitro eNOS activation was also recorded, thus FGR placentas had a decreased eNOS/arginase-2 ratio, which may have been the contributing factor to relative vasoconstriction of the vascular beds. Another study has demonstrated that the expression of both ARG2 and eNOS are independently regulated by DNA methylation and histone post-transcriptional modifications in HUAECs [98,99]. eNOS activity in the endothelium is also believed to be regulated by the expression of dimethylarginine dimethylaminohydrolase 1 (DDHA1) and nuclear factor-erythroid factor 2-related factor 2 (NRF2) [100,101,102]. One of the eNOS endogenous inhibitors, asymmetric dimethylarginine (ADMA), is metabolized by DDHA1 Thus, an increased DDHA1/ADMA ratio is associated with endothelial protection (Figure 4) [103]. NRF2 also protects the endothelium by acting as a transcription factor which induces the expression of SOD1 and GPX1, which in turn promotes antioxidant response.

#### 3.2.2. Endothelial Function in FGR Resulting from PE Compared to FGR in Normotensive Pregnancies

Studies have traditionally attempted to determine whether the mechanism of endothelial dysfunction and hypoxia resulting from PE is similar to that in normotensive patients with FGR. A recent study analyzed the relationship between endothelial function and oxidative stress in normotensive women with FGR, compared to those with PE and normal pregnancies, by measuring concentrations of oxygen free radicals (d-ROMs), PlGF, sFlt-1 and placental oxidative damage [104]. Once the placenta becomes hypoxic, the production of free radicals and hypoxia-inducible factor-1α (HIF-1α) takes place in placenta decidua [105,106]. As a result of HIF-1α overexpression, sFlt-1 is upregulated, which promotes a cascade of events leading to angiogenesis. sFlt-1 serves as a receptor for VEGF and PlGF. However, once sFlt-1 is present in excess, VEGF and PlGF production by placenta is suppressed, resulting in maternal endothelial dysfunction [104]. Although the maternal serum concentration of sFlt-1 was increased, while that of PlGF was decreased in patients with early- or late-onset PE, levels of those factors in patients with normotensive FGR remained comparable to those with normal pregnancies. Not surprisingly, the sFlt-1/PlGF ratio has been used as a clinical tool to rule out the onset of PE in women with suspected disease [107]. At the same time, higher than normal placental DNA oxidative damage, measured by the portion of nuclei staining positive for 8-OHdG, was recorded in both patients with PE and FGR. Thus, while oxidative stress was evident in both groups, a different mechanism of oxidative stress induction (irrespective of sFlt-1 signalling) may be implicated in patients with normotensive FGR. For instance, maternal plasma soluble endoglin, a factor inhibiting angiogenesis, was found to be significantly increased in women with normotensive FGR between the first and second trimesters. Additionally, an increase in expression of transforming growth β-induced factor was reported in FGR placentas in two mice studies [108,109]. This factor hinders transcriptional activation of TGF- β, which is normally involved in placental angiogenesis, cell proliferation, differentiation and apoptosis.

#### 3.2.3. Transcriptional Modification in FGR Placentas

Additionally, certain miRNAs, especially miR-21 and miR-126, were responsive to hypoxia in HUVECs [110]. These miRNAs have been involved in angiogenesis, the regulation of endothelial function, and vascular remodeling. In the context of FGR, one study found hsa-miR-21 to be decreased and miR-126 to be elevated in cultured FGR HUVECs [111]. While a decrease in miR-21 was attributed to hg-miR-21 gene promoter methylation, no changes to miR-126 promoters were observed in the study. There was a reverse relationship observed between the levels of miR-21 and those of eNOS and DDHA1, and further analysis revealed the decreased stability of eNOS and DDHA1 transcripts in the presence of this miRNA subtype. It was concluded that the selective expression of the hypoxic-miRNA profile in FGR plays a role in the regulation of the NO pathway in endothelial cells.

#### 3.2.4. Long-Term Clinical Implication of FGR Pregnancies

Overall, endothelial dysfunction implicated in FGR pregnancies has debilitating long-lasting effects on both the mother and the fetus [84]. Neonates born in the aftermath of FGR are not only at increased risk for perinatal morbidity and mortality, but are also more likely to experience systemic hypertension (HTN), coronary heart disease, atherosclerosis, and chronic kidney disease (CKD). According to several animal models, FGR pregnancies were at increased risk of developing HTN later in life [112,113]. Human epidemiological study results are also in accordance with these findings, reporting a correlation between increased blood pressure and low body weight in infancy, adolescence and adulthood. Moreover, a recent study reports a direct link between hypertension and low birth weight. Endothelial dysfunction has also been associated with CKD. In fact, an analysis of 18 studies reports that infants born after FGR are at a higher risk of developing albuminuria, end-stage renal disease and a decreased glomerular filtration rate later in life [114]. However, the sequence of events remains undetermined, therefore it is unclear whether endothelial dysfunction precedes the development of both HTN and CKD [84]. After FGR pregnancies, women may experience cardiovascular system dysfunction in the form of asymptomatic heart failure, the increased risk of developing hypertension, and an increased risk of mortality from cardiovascular-related illness [115].

### 3.3. Endothelial Dysfunction in Pregnancies with Diabetes

Diabetes results in a risk of developing various pregnancy-related complications, such as PE, preterm delivery, or placental insufficiency. Numerous studies have attempted to identify an elusive multifactorial pathogenesis of those adverse pregnancy outcomes, which are undoubtedly associated with vascular dysfunction. It is believed that the increased fetomaternal morbidity and mortality observed in patients with diabetes is linked with endothelial damage induced by both hyperglycemia and chronic low-grade inflammation [116,117].

#### 3.3.1. Markers of Endothelial Dysfunction in Diabetes

##### Adhesion Molecules

Several membrane proteins, such as VCAM-1, intercellular adhesion molecule 1 (ICAM-1), or E-selectin represent the group of biomarkers characteristic of endothelial cells [118]. These adhesion molecules mediate the interactions with the cells mediating the inflammatory process. Endothelial activation increases the expression of adhesion molecules and promotes the attachment and migration of adherent monocytes, which precede the endothelial damage [119,120]. Significantly increased serum concentrations of soluble VCAM-1 have been noted in patients with gestational diabetes mellitus (GDM) and type 1 diabetes (T1D) compared with the control group [121,122,123,124]. Furthermore, the prolonged increase in concentrations of adhesion molecules in the peripheral blood were noted in patients with diabetes progression after pregnancy and in women with a history of GDM [118,125,126]. In contrast to those findings, Bajaj et al. noted markedly decreased VCAM-1 levels in the group of women with GDM three years after pregnancy [127]. Finally, others discovered no significant differences in the VCAM-1 and ICAM-1 concentrations in maternal blood when comparing GDM patients and controls [128,129,130,131]. Interestingly, Diaz-Perez et al. found significantly decreased ICAM-1 protein concentrations in patients with GDM, while there were no changes in the ICAM-1 mRNA expression. Moreover, they found a negative correlation between the ICAM-1 protein and maternal body mass index (BMI). Thus, they hypothesized that decreased ICAM-1 in patients with GDM could be connected with the hypothetical protective post-translational regulations [130]. In contrast to those findings, other studies detected increased ICAM-1 levels in patients with GDM and T1D [122,123,124,132,133,134]. The increased ICAM-1 expression in decidual endothelial cells collected from women with T1D was associated with higher monocyte adhesion, which was reduced following the ICAM-1 antibody blockade [135]. Finally, it was discovered that the adhesion molecules’ concentrations might be associated with fetal growth disturbances in patients with pregestational diabetes [136].

##### Other Mediators of Endothelial Dysfunction

It has been speculated that the disturbances in the expression of several proteins involved in the regulation of angiogenesis could play a role in the development of various pregnancy-related complications. Lappas revealed that there were no differences in the placental expression and secretion of adhesion molecules (VCAM-1, ICAM-1) and the regulators of angiogenesis (i.a., PIGF, VEGF, sFlt-1, and fibroblast growth factor 2 [FGF2]) in patients with GDM [134]. However, significant changes in the expression of numerous proteins associated with endothelial damage have been discovered in the explants of omental adipose tissue. Du et al., in their prospective longitudinal study, assessed the secretion of serum inflammatory markers in women with T1D who developed PE and those who remained normotensive. They revealed significant differences in the C-reactive protein (CRP), E-selectin, interferon-γ-inducible protein-10, interleukin-1 (IL-1) receptor antagonist, and eotaxin serum concentrations [137]. Also, ADMA, which acts as an inhibitor of eNOS, has been investigated as a potential marker of endothelial dysfunction in GDM. However, there are numerous contradictory results of ADMA measurements between various studies [131,132,138,139,140]. Tekin et al. hypothesized that alterations in plasma levels of signal peptide-CUB-EGF domain-containing protein (SCUBE)-1, which was found to be associated with endothelial dysfunction, could also be detected in patients with GDM. They discovered that SCUBE-1 concentrations were markedly increased in patients with GDM. However, the clinical relevance of these findings should be further tested [141]. Hiden et al. found increased membrane-type matrix metalloproteinase 1 (MT1-MMP) protein expression in placentas obtained from patients with GDM. They revealed that MT1-MMP expression in primary fetoplacental endothelial cells is stimulated by insulin and insulin-like growth factor 2 (IGF-II) via phosphatidylinositol 3-kinase (PI3K) through insulin receptors. Moreover, MT1-MMP blocking reduced angiogenesis in vitro [142].

#### 3.3.2. Functional Assessment of Endothelial Damage In Vivo

Numerous studies have assessed the vascular function in pregnant women with diabetes. Bugatto et al. found that the mean uterine artery Doppler pulsatility index in patients with GDM was significantly positively correlated with IL-6, triglycerides and glycated hemoglobin, implying that alterations in lipids and glycemic homeostasis are associated with vascular pathologies [128]. For instance, placental atherosclerosis was discovered significantly more often in patients with GDM compared with normal pregnancies [143]. Hence, carotid artery intima-media thickness (IMT) measurement is used to assess subclinical atherosclerosis and predict the risk of future adverse cardiovascular events in asymptomatic patients [144]. Atay et al. found that normotensive patients with GDM had significantly increased IMT and homocysteine concentrations, as well as reduced nitric oxide levels [145]. Moreover, markedly increased IMT was detected in women with GDM in a previous pregnancy, 6.5 years after delivery, compared with the control group. Interestingly, IMT values were significantly associated with E-selectin, ICAM-1, IL-6, and CRP serum concentrations [126]. These observations imply the association between GDM and endothelial damage. When measuring endothelial function with FMD, patients with GDM had significantly reduced values compared with healthy controls [146,147]. Moreover, FMD reduction in women with GDM persisted in the early postpartum period [148]. Furthermore, the increased arterial stiffness, indicated by lower distensibility of the brachial and carotid artery, was noted in patients with GDM [148]. Mrizak et al. measured the forearm skin blood flow (FSBF) in response to acetylcholine in patients with GDM and the control group. They found significantly reduced FSBF values in the GDM group [149]. It was reported that myometrial arteries collected from patients with GDM had markedly impaired endothelium-dependent relaxation ex vivo compared with the control group [150]. Those findings are consistent with the thesis that GDM is connected with vascular damage. In contrast, Acosta et al. found no differences in vascular reactivity measured by laser Doppler examination in patients with GDM and control individuals [151]. Moreover, Ang et al. did not detect any alterations in either endothelial or smooth muscle function in small arteries obtained from patients with T1D [152]. Endothelial dysfunction could result in imbalances in the production of vasoconstrictor and vasodilator molecules. Nonetheless, Swiderski et al. found no differences in the serum endothelin-1 and cyclic guanosine monophosphate concentrations in patients with GDM, pregestational diabetes, and healthy study participants [153]. Furthermore, few studies assessed the vascular function several years postpartum. Banerjee et al. reported that maximal endothelium-dependent dilation related to carbachol was reduced in patients with GDM (individuals previously enrolled in the HAPO study) [154]. They found that inhibition of eNOS did not affect arteries dilation in patients with GDM, which suggests the impairment of eNOS activity associated with hyperglycemia [154]. There were no significant alterations in FMD values in patients with a history of GDM and women with normal glucose tolerance six years postpartum [155]. Khurana et al. reported that while hyperinsulinemia did not change FMD values, hyperglycemia may reduce it in the late third trimester in patients with type 2 diabetes [156]. It was reported that pregnancy improves microvascular reactivity, measured by laser Doppler imaging and iontophoretic administration of endothelial-dependent and endothelial-independent vasodilators, compared to the postnatal period in women with T1D. Nonetheless, control pregnant patients experienced markedly higher endothelial function enhancement than T1D patients [152]. Interestingly, there were no significant differences in skin microvascular reactivity, determined using laser Doppler fluximetry, in patients with GDM and healthy controls [157].

#### 3.3.3. Diabetes, Preeclampsia and Other Pregnancy-Related Complications

Kul et al., in their comparative study, reported that most of the patients with a history of PE and GDM (91%) present features of coronary microvascular dysfunction, defined by coronary flow reserve measured in echocardiography. Interestingly, the prevalence of microvascular dysfunction is significantly lower in patients with isolated GDM (55%) compared with individuals with combined PE and GDM [158]. Moreover, the increased sFlt1/PIGF ratio, pronounced by the overexpression of anti-angiogenic sFlt1, is linked with the development of PE in the general population. Similar findings have been noted in pregnant women with GDM [159]. It is believed that the altered adhesion molecules’ expression could play a role in the development of PE. Clausen et al. discovered that elevated plasma ICAM-1 and VCAM-1 concentrations are detected in the late first trimester in women with T1D who developed PE in latter pregnancy [124]. ACE gene I/D polymorphism is associated with increased PE risk in the general population. Dmitrenko et al. postulated that this polymorphism also elevates the risk of PE in patients with GDM [160].

**Figure 5:**
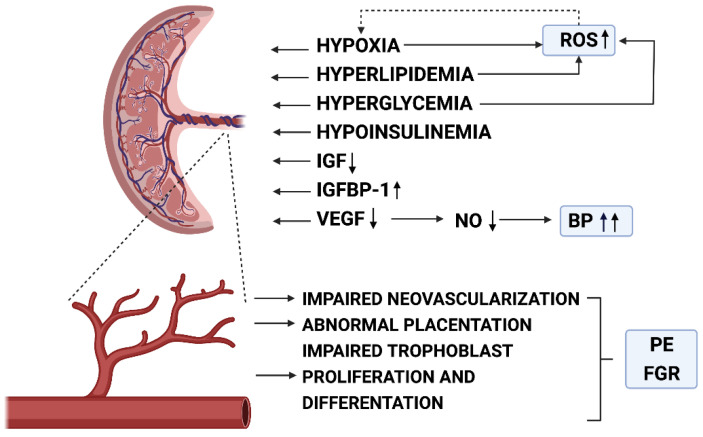
Pathomechanisms of vascular injury, hypertension and abnormal placentation in pregnancy with diabetes. BP-blood pressure; FGR-fetal growth restriction; IGF-insulin-like growth factor; IGFBP-1-insulin-like growth factor-binding protein-1; NO-nitric oxide; PE-preeclampsia; ROS-reactive oxygen species; VEGF-vascular endothelial growth factor. Created with Biorender.com (accessed on 19 October 2021).

Pre-existing diabetes (T1D, or T2D) exerts more negative consequences for feto-maternal vasculature in comparison to GDM. Prolonged maternal hyperglycemia induces multiple pathological processes (i.e., non-enzymatic glycosylation, alterations in lipid metabolism, local hypoxia, increased ROS synthesis, imbalances in cytokines and growth factors production) that trigger vascular remodeling and endothelial injury. As a result, patients with long-lasting diabetes are significantly more predisposed to PE development compared to women with GDM [161] (Figure 5).

Gutaj et al. reported that primiparity and diabetic vasculopathy were the strongest predictors of PE in women with T1D. Moreover, they found that pregestational hypertension, high gestational weight gain, and increased HbA1c and triglyceride concentrations in maternal blood were also associated with the elevated risk of PE [162]. Pregnancies of patients with T1D are also frequently complicated by disturbances in fetal growth. Zawiejska et al., investigating the concentrations of markers of endothelial injury in maternal blood, revealed that both excessive and small-for-gestational-age (SGA) fetal growth might be associated with diabetes-induced endothelial dysfunction [136]. Furthermore, Gutaj et al. noted that the low PlGF serum levels in the mid-pregnancy, as well as no increase in its values from early to mid-pregnancy, may be potentially indicative of SGA in patients with T1D [163]. Our 25 years of experience in treating patients with long-duration T1D proves that early introduction of optimal treatment measures and education on the role of strict glycemic control and lifestyle habits significantly reduces the risk of adverse pregnancy outcomes in patients with T1D [164].

#### 3.3.4. Assessment of Endothelial Function in In Vitro Conditions

Maternal exposure to hyperglycemia is connected with increased fetal vascular resistance. It was found that those changes are associated with reduced phosphorylation of eNOS and protein kinase B (AKT) at key residues involved in the nitric oxide synthesis [165]. Anaya et al. found that the HUVECs isolated from mothers with diabetes showed disrupted Ca^2+^ bursts which resulted in impaired activation of eNOS and reduced NO production that could contribute to the development of vascular pathologies in patients with diabetes [166]. However, Mordwinkin et al. detected the increased eNOS expression in both maternal and cord blood [123]. Furthermore, HUVECs isolated from patients with GDM show features of decreased proliferation, migration, and formation of the new vessels compared to those obtained from healthy individuals [167,168,169,170,171]. It was reported that hyperglycemia inhibits the proliferation of HUVESCs stimulated by FGF2 but did not change the vascular endothelial growth factor-induced angiogenesis, which is controlled by extracellular signal-regulated kinases 1/2 (ERK1/2) [169]. Cvitic et al. found significant variation in the DNA methylation patterns and expression of genes attributed to cell morphology and cellular movement in both arterial and venous fetoplacental endothelial cells obtained from women with GDM and healthy controls [172]. Moreover, Blue et al., using the genome-wide mRNA expression analysis, revealed that endothelial dysfunction is associated with alterations in gene expression and DNA methylation [173]. Prieto et al. reported that Netrin-1 and its receptors might regulate the increased placental angiogenesis observed in GDM. They found the decreased expression of anti-angiogenic Unc-5 Netrin Receptor B in HUVECs obtained from patients with GDM [174]. In turn, Peng et al. discovered increased miR-137 plasma levels in women with GDM and HUVECs exposed to hyperglycemia. They reported that elevated miR-137 expression is associated with decreased cell viability and angiogenesis, and increased secretion of inflammatory cytokines and monocytes adhesion to HUVECs [175]. Floris et al. proposed a theory that describes the role of miR-101 upregulation in the pathogenesis of HUVECs damage in patients with GDM. They speculated that the inhibition of miR-101 increased the enhancer of zester homolog-2 (EZH2) expression and possibly improved the HUVECs’ bioactivity [171]. Meanwhile, Ye et al. investigated the role of maternally expressed gene 3 (MEG3) in the development of endothelial dysfunction. They revealed that hyperglycemia promotes the expression of the MEG3 gene that could be partially responsible for endothelial dysfunction triggered by hyperglycemia. They found that MEG3 downregulated miR-370-3p and stimulated overexpression of the AFF1 gene by inhibition of the PI3K/AKT pathway and, consequently, played a role in endothelial damage [167]. Alqudah et al. found a significant reduction in the FK506-binding protein like (FKBPL) and the upregulation in the PIGF and vascular endothelial growth factor receptor 1 (VEGF-R1) expression in placental tissue samples collected from women with T1D. In contrast to those observations, only the expression SIRT-1 gene was significantly downregulated in patients with GDM compared to the controls [176]. Moreover, the high FKBPL expression in HUVECs, evoked by plasmid vector transfection, was associated with a reduction in tubule formation and could be potentially responsible for endothelial dysfunction in vivo [176]. Di Tomo et al. reported that HUVECs collected from women with GDM presented alterations in mitochondrial membrane potential and antioxidant response compared with cells obtained in controls. Endothelial cells obtained in patients with diabetes exhibited a reduced SIRT-1 expression and increased p16, p21, and p53 activity. They postulated that those changes, together with p300 activation, induce persistent endothelial senescence in patients with GDM [177]. A microarray analysis revealed significant differences in the expression of numerous genes involved in cellular function and proliferation regulation in HUVECs collected from healthy controls and patients with GDM. Moreover, the same study found differences in cell cycle distribution in those cellular subpopulations—HUVECs from women with GDM had an increased proportion of cells in the G2/M phase, indicating the immobilization of cell divisions provoked by prolonged hyperglycemia. All of those factors and increased mitochondrial superoxide generation may contribute to endothelial dysfunction pathogenesis [178]. Saez et al. investigated the influence of hyperglycemia on exosomes secretion and bioactivity in HUVECs [179,180,181]. They found that hyperglycemia stimulated the exosome release. Furthermore, exosomes isolated from HUVECs cultured under hyperglycemic conditions promoted endothelial cell wound healing as well as the expression of phosphorylated endothelial nitric oxide synthase, human cationic amino acid transporter type 1 (hCAT-1), and ICAM-1 in HUVECs cultured in normal glucose concentrations, imitating the destructive effects of hyperglycemia [179]. Results observed in patients with T2D are consistent with findings from GDM studies—HUVECs obtained from T2D patients presented with lower proliferation rates, increased apoptosis, and higher levels of superoxide anions [182]. Moreover, Sultan’s study identified 132 genes, expression of which was significantly altered in umbilical endothelial cells obtained from patients with T2D [182].

#### 3.3.5. Potential Inhibitors of Endothelial Dysfunction in Diabetes

Interestingly, it was discovered that myo-inositol supplementation could restrict endothelial dysfunction in women with GDM. HUVECs obtained from women with GDM who were treated with myo-inositol throughout the pregnancy had a markedly reduced number of monocytes attached to their surface, less adhesion molecule exposure, and lower intracellular oxidative stress levels in comparison to those of women treated with diet only. The same effects were observed after 48h hours of stimulation with myo-inositol in in vitro conditions [183]. Another in vitro study revealed that metformin improves cell migration and stimulates angiogenesis of HUVECs. It was speculated that both processes are promoted by the increased NRF2 and downregulated p65 expression caused by the metformin treatment [184]. Liraglutide significantly reduced the monocyte adhesion, the expression of adhesion molecules, MAPK/NF-kB activation, peroxynitrite levels, and endothelial microvesicle release in the HUVECs exposed to tumor necrosis factor-alpha (obtained from patients with GDM) [185]. Furthermore, Hemling et al. found that the thioredoxin mimetic peptides supplementation improves migration, proliferation, survival and restores VEGF resistance in HUVECs [186]. Based on the in vitro observations, the carotenoid-rich diet may exert protective effects on the HUVECs in diabetic conditions [187]. Also, the purified Ovothiol A exerted anti-inflammatory effects (decreased the expression of adhesion molecules and monocyte-HUVEC interaction) and reduced the oxidative stress in HUVECs in vitro [188]. Gui et al. discovered that vitamin D supplementation could restore the features of endothelial dysfunction in vitro in endothelial colony-forming cells obtained from cord blood in women with GDM [170]. Also, the Centella Asiatica and lipoic acid treatment inhibits the monocyte adhesion to HUVECs in in vitro conditions [189]. Interestingly, it was reported that the human chorionic mesenchymal stem cells co-cultured with the HUVECs could reverse the high glucose-induced endothelial damage and restore cell functionality [190]. Moreover, a conditioned medium of human embryonic stem cell-derived endothelial cells could reverse endothelial progenitor cell dysfunction in patients with T2D [191]. Subiabre et al. discovered that maternal insulin therapy does not restore the biological functions of fetoplacental endothelial cells in patients with GDM [192]. However, insulin therapy acts differentially in HUVECs collected from patients with GDM and healthy controls [193]. Therefore, it could restore the reduced adenosine uptake via human equilibrate nucleoside transporter 1, -2 (hENT1, hENT2) [194,195].

## 4. Conclusions

In the review we presented the role of endothelial dysfunction and endothelial injury in three pathologies of pregnancy: PE, FGR and diabetes, which often coexist with clinical situations, sparking the need for their precise differentiation.

Overall, the endothelium plays an important role in maintaining blood vessel integrity. There are theories linking alterations in endothelial integrity and patomechanism of certain gestational conditions, including PE, FGR and DM, during pregnancy. However, the clear differentiation of the involved mechanisms is challenging, because these complications often coexist. The future of the research lies in fine tuning the clinical markers to be used and identifying possible therapeutic techniques to reduce endothelial injury, improve its function or promote regenerative mechanisms.

FGR without PE is usually not associated with a significant elevation of serum markers of endothelial injury like sVCAM-1 or HA, which are increased in cases of PE with or without FGR. Furthermore, both gestational diabetes and PE are associated with the increased concentrations of some markers of endothelial dysfunction. However, the mechanism of endothelial injury in PE is probably different and depends more on the elevation of sFlt-1 levels. The use of antiangiogenic markers, including sFlt-1 and placental growth factor (PlGF), rather than markers of endothelial dysfunction, may be helpful to differentiate some cases of PE from cases of exacerbation of diabetic kidney disease in pregnancy.

## Data Availability

Not applicable.
